# Interleukin-8 Responses of Multi-Layer Gingival Epithelia to Subgingival Biofilms: Role of the “Red Complex” Species

**DOI:** 10.1371/journal.pone.0081581

**Published:** 2013-12-10

**Authors:** Georgios N. Belibasakis, Thomas Thurnheer, Nagihan Bostanci

**Affiliations:** 1 Oral Microbiology and Immunology, Institute of Oral Biology, Center of Dental Medicine, University of Zürich, Zürich, Switzerland; 2 Oral Translational Research, Institute of Oral Biology, Center of Dental Medicine, University of Zürich, Zürich, Switzerland; Charité-University Medicine Berlin, Germany

## Abstract

Periodontitis is an infectious inflammatory disease that results in the destruction of the tooth-supporting (periodontal) tissues. The Gram-negative anaerobic species *Porphyromonas gingivalis*, *Tannerella forsythia* and *Treponema denticola*, (also known as the “red complex” species) are highly associated with subgingival biofilms at periodontitis-affected sites. A major chemokine produced by the gingival epithelium in response to biofilm challenge, is interleukin (IL)-8. The aim of this *in vitro* study was to investigate the relative effect of the “red complex” species as constituents of subgingival biofilms, on the regulation of IL-8 by gingival epithelia. Multi-layered organotypic human gingival epithelial cultures were challenged with a 10-species *in vitro* subgingival biofilm model, or its 7-species variant, excluding the “red complex”. IL-8 gene expression and secretion analyses were performed by qPCR and ELISA, respectively. After 3 h, both biofilms up-regulated IL-8 gene expression, but the presence of the “red complex” resulted in 3-fold greater response. IL-8 secretion was also up-regulated by both biofilms, with no differences between them. After 24 h, the 10-species biofilm reduced IL-8 secretion to 50% of the control, but this was not affected when the “red complex” was absent. In conclusion, as part of biofilms, “red complex” species differentially regulate IL-8 in gingival epithelia, potentially affecting the chemotactic responses of the tissue.

## Introduction

Periodontal diseases are infectious inflammatory diseases, caused by endogenous oral bacteria colonizing the tooth surfaces as polymicrobial biofilm communities. Interaction of biofilms with the juxtaposing periodontal tissues triggers an inflammatory response, aiming to prevent bacterial colonization and establishment [1]. Nevertheless, an excessive inflammatory response will result in periodontal tissue destruction, manifesting as periodontitis, and tooth loss, if this condition is left untreated [2]. Periodontitis is attributed to the establishment of a “subgingival” biofilm, consisting of characteristic bacterial species. The presence of the tree “red complex” species, namely *Porphyromonas gingivalis*, *Treponema denticola* and *Tannerella forsythia*, in subgingival biofilms has been highly associated with periodontitis [3].

The epithelium of the gingival sulcus or periodontal pocket is the first line of defence against the developing biofilm, by constituting a physical barrier and supporting the trafficking of polymorphonuclear neutrophils through it, which tackle the establishment of biofilm infection [4]. By producing chemokines, the epithelial cells drive the recruitment of neutrophils through the epithelial layers into the affected area of the sulcus. Interleukin (IL)-8 is the best characterized chemokine, with a crucial role in clinically healthy junctional epithelium, as it presents a gradient for neutrophil recruitment in the gingival crevice [5]. IL-8 expression and production is induced in a number of cell types by *T. denticola* [6], [7], *T. forsythia* [8], [9], or *P. gingivalis* [9], [10], as well as a mixed infection of all three species [11]. It has also been demonstrated that single species biofilms are more effective in inducing IL-8 production by oral epithelial cells than their planktonic counterparts, with the exception of *P. gingivalis* [12]–[14], especially when part of a three-species biofilm [15]–[17]. A 9-species biofilm including *P. gingivalis* and *T. forsythia* induced IL-8 production in human gingival epithelial cell cultures [18]. Nevertheless, a number of studies are contrasting this trend, by demonstrating lack of effect or even inhibition of IL-8 production by epithelial cells in response to *T. denticola* [19]–[21], or *P. gingivalis* [19], [22], [23]. The capacity of *P. gingivalis* in planktonic state to inhibit IL-8 gene expression by gingival epithelial cells has been designated as “chemokine paralysis” [22].

It therefore appears that the available data on the role of the “red complex” species in IL-8 regulation of by gingival epithelial cells is rather conflicting. The aim of this study was to investigate the effect of a 10-species *in vitro* subgingival biofilm model on the expression and secretion of IL-8 by multi-layered tissue-like gingival epithelium, and to evaluate the relative involvement of the three “red-complex” species as a specialized bacterial community within the biofilm. The uniqueness of the employed experimental model is that it closely resembles the *in vivo* conformation of a multispecies subgingival biofilm, juxtaposed against the multi-layered gingival epithelium. Hence, it is highly relevant for studying host-responses, such as IL-8 in the present study.

## Materials and Methods

### *In vitro* Biofilm Model

The 10-species *in vitro* “subgingival” Zürich biofilm model [18], [24] used in this study, consisting of *Campylobacter rectus* (OMZ 697), *Fusobacterium nucleatum* (OMZ 596), *P. gingivalis* ATCC 33277^T^ (OMZ 925), *Prevotella intermedia* ATCC 25611^T^ (OMZ 278), *T. forsythia* OMZ1047, *T. denticola* ATCC 35405^T^ (OMZ 661), *Veillonella dispar* ATCC 17748^T^ (OMZ 493), *Actinomyces oris* (OMZ 745), *Streptococcus anginosus* (OMZ 871), and *Streptococcus oralis* SK 248 (OMZ 607). A 7-species variant of this biofilm was also grown, in the absence of *P. gingivalis*, *T. forsythia* and *T. denticola*. Briefly, the biofilms were grown in 24-well cell culture plates on sintered hydroxyapatite discs, resembling a natural tooth surface. To achieve pellicle formation, these surfaces were pre-conditioned for 4 h with 800 µl of human saliva diluted 1∶1 in sterile saline. To initiate biofilm formation, the hydroxyapatite discs were covered for 16.5 h with 1.6 ml of growth medium consisting of 60% saliva, 10% heat-inactivated human serum, 30% modified fluid universal medium (mFUM) [25], [26] containing 0.3% glucose, and 200 µl of a bacterial cell suspension containing equal volumes and densities from each strain. After 16.5 h of anaerobic incubation at 37°C, the medium was replenished, and 50 µl of *T. denticola* liquid culture were also added (OD_550_ = 1.0). Biofilms were grown anaerobically for further 48 h and during this period, the discs were “dip-washed” in saline three times daily for 1 min, and the medium was replenished once daily.

After a total of 64.5 h, the biofilm-carrying hydroxyapatite discs were carefully placed onto multi-layered gingival epithelial cell cultures (described below), mediated by a plastic ring to ensure a distance of 1 mm. As matched controls, pellicle pre-coated hydroxyapatite discs were used that did not contain grown biofilm cultures. The exposure of the cell cultures to the biofilms lasted for 3 h or 24 h. At each time-point, the discs were removed from the cultures and subsequently processed for analysis of bacterial composition by quantitative real-time Polymerase Chain Reaction (qPCR), as previously described [25]. The cell culture supernatants as well as the multi-layered epithelial cells were further processed.

### Cell Cultures and Cytotoxicity Assay

Stratified gingival organotypic epithelial cell cultures in 24-well plate format (0.5 cm^2^ surface) were used (EpiGing, MatTek, Ashland, MA, USA) and maintained in culture in defined keratinocyte serum-free medium (K-SFM), supplemented with 0.05 mM calcium chloride and 200 mM L-glutamine (Gibco/Invitrogen, Lucerne, Switzerland). This model consists of normal human gingival epithelial cells cultured to form a highly differentiated multi-layered tissue with keratinized layers, that resembles morphologically the gingival epithelium. This *in vitro* tissue expresses cytokeratin K13 and K14, as well as human beta defensins (HBD) HBD-1 and HBD-3. For the experiments, these gingival tissue cultures were brought in co-culture with the biofilms described in the previous section, for 3 h or 24 h.

The potential cytotoxic effect of the biofilms on these cultures was evaluated by measuring the extracellularly released lactate dehydrogenase (LDH) activity, over 24 h, using the CytoTox 96 Non-radioactive Cytotoxicity Assay (Promega, Mannheim, Germany).

### RNA Extraction and cDNA Synthesis

Upon completion of the experiments, the culture supernatants were removed and the multi-layered gingival epithelia were washed twice in PBS, before being lysed. Total RNA was extracted by the RNeasy Mini Kit (QIAGEN, Hombrechtikon, Switzerland), and its concentration was measured by a NanoDrop spectrophotometer. One µg of total RNA was then reverse transcribed into single-stranded cDNA by M-MLV Reverse Transcriptase, Oligo(dT)_15_ Primers, and PCR Nucleotide Mix (Promega, Mannheim, Germany), at 40°C for 60 min, and 70°C for 15 min. The resulting cDNA was stored at −20°C.

### Gene Expression Analysis by qPCR

Gene expression analysis was performed by qPCR, in an ABI Prism 7000 Sequence Detection System and software (Applied Biosystems, Carlsbad, CA). For the amplification reactions, TaqMan Gene Expression Master Mix and Gene Expression Assay kits (Applied Biosystems, Life Technologies, Zug, Switzerland) were used (assay IDs: IL-8: Hs00174103-m1, GAPDH: Hs99999905-m1). The standard PCR conditions were 10 min at 95°C followed 40 cycles at 95°C for 15 seconds and 60°C for 1 min. The expression levels of the target transcripts in each sample were calculated by the comparative Ct method (2^−ΔCt^ formula), after normalization to GAPDH (housekeeping gene).

### Measurement of IL-8 by Enzyme-linked Immunosorbent Assay (ELISA)

The concentrations of IL-8 secreted by into the culture supernatant were evaluated by a commercially available ELISA kit (DY208, Duo Set Human CXCL8/IL-8 ELISA, R&D Systems, Abingdon, UK). Absorbance was measured at 450 nm, with wavelength correction of 570 nm, using a microplate reader (Epoch, BioTek, Lucerne, Switzerland). A standard curve was created using known concentrations of rhIL-8 provided in the kit. The concentration of IL-8 in each sample was calculated by a four-parameter logistic (4-PL) equation. The sensitivity of the assay was 15 pg/ml.

### Statistical Analysis

One-way or two-way analysis of variance (ANOVA) was used to analyze statistical differences, using Bonferroni or Sidak’s multiple comparisons tests, respectively. Differences were considered statistically significant at *P*<0.05.

## Results

The multi-layered gingival epithelial cultures were challenged for 3 h and 24 h with either the 10-species subgingival biofilm, or its 7-species variant, which excluded the “red complex” species. The bacterial composition of the two biofilms was investigated at these time points. It was found that the absence of the three “red complex” species did not significantly affect the quantitative composition of the remaining individual species by more than 1-log, neither after 3 h nor after 24 h in culture (Table 1). Moreover, there were no statistically significant differences (*P*>0.05) in released the LDH levels between groups (Table 2), and hence neither of the biofilms was able to induce cytotoxic effects during this challenge period.

**Table 1:** Bacterial composition of the subgingival biofilms after 3

*3 h*	*10-species*	*7-species (- Red Complex)*
***A. oris***	1.6 E7±2.2 E7	1.5 E7±7.1 E6
***V. dispar***	3.8 E7±3.4 E7	6.0 E7±3.3 E7
***F. nucleatum***	3.4 E8±4.1 E8	2.3 E8±6.5 E7
***S. anginosus***	3.5 E8±4.0 E8	2.4 E8±7.4 E7
***S. oralis***	1.1 E9±1.4 E9	6.1 E8±2.2 E8
***P. intermedia***	1.1 E9±1.2 E9	7.5 E8±2.0 E8
***C. rectus***	7.7 E6±1.3 E6	4.9 E6±4.9 E5
***P. gingivalis***	1.6 E7±2.0 E7	–
***T. denticola***	4.1 E7±3.0 E7	–
***T. forsythia***	7.6 E6±2.2 E6	–
***24 h***	***10-species***	***7-species (- Red Complex)***
***A. oris***	8.3 E6±6.4 E6	4.6 E6±1.1 E6
***V. dispar***	2.1 E7±1.7 E7	2.0 E7±4.4 E6
***F. nucleatum***	8.7 E7±8.4 E7	1.0 E8±5.3 E7
***S. anginosus***	2.0 E8±1.6 E8	5.3 E7±2.5 E7
***S. oralis***	5.8 E8±5.9 E8	2.1 E8±1.4 E8
***P. intermedia***	4.2 E8±3.2 E8	2.3 E8±1.0 E8
***C. rectus***	4.6 E6±2.5 E6	2.2 E6±7.0 E5
***P. gingivalis***	2.2 E6±3.7 E6	–
***T. denticola***	1.6 E7±1.0 E7	–
***T. forsythia***	8.3 E6±6.3 E6	–

The numerical composition of the individual bacterial species in the biofilms after 3 h and 24 h in co-culture with the multi-layered gingival epithelia was evaluated by quantitative real-time polymerase chain reaction (qPCR). The values represent bacterial numbers (mean ± SEM), from three independent biofilms in each group. Two-way ANOVA was used to calculate the differences between groups (*P*<0.05).

**Table 2:** Cytotoxic effects in response to the subgingival biofilm challenge.

	*Control*	*10-species*	*7-species (- Red Complex)*
**3 h**	0.82±0.08	0.76±0.12	0.60±0.03
**24 h**	0.70±0.04	0.73±0.04	0.86±0.21

The values represent the extracellulalry released LDH activity (mean OD ± SEM) measured spectophotometrically, from three independent cell cultures in each group. One-way ANOVA was used to calculate the differences between groups (*P*<0.05).

**Figure 1:**
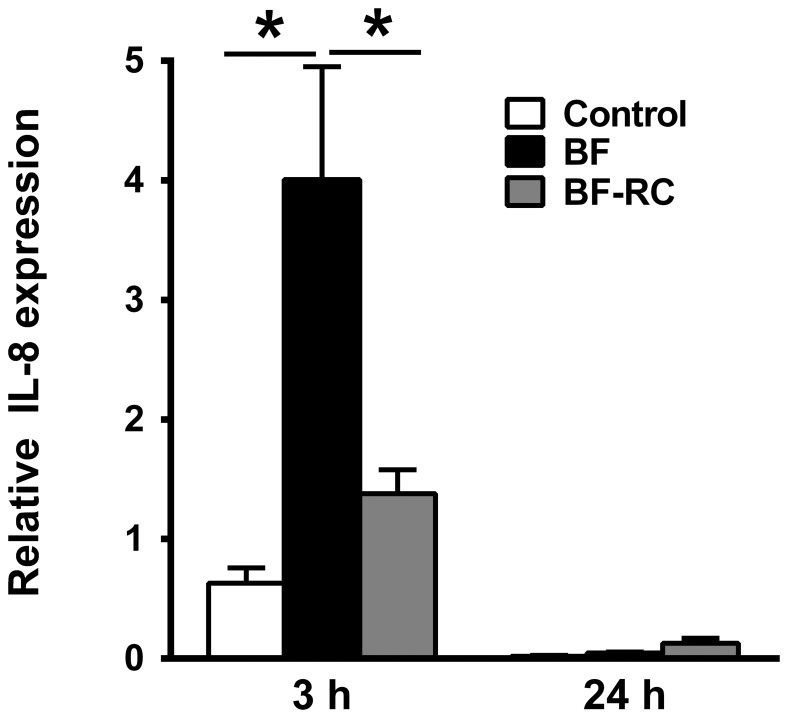
Regulation of IL-8 gene expression in response to biofilm challenge. Multi-layered gingival epithelial cell cultures were challenged for 3 h or 24 h with the 10-species or 7-species (excluding *P. gingivalis*, *T. forsythia*, *T. denticola*) subgingival biofilm. IL-8 gene expression was measured by qPCR calibrated against GAPDH, and expressed as the 2^−ΔCT^ formula. Bars represent mean values ± SEM from three independent cell cultures in each group. One-way ANOVA was used to calculate the differences between groups. Asterisks represent statistically significant differences between groups (*P*<0.05).

The effect of these two biofilms variants on the gene expression of the IL-8 was then investigated. After 3 h, the 10-species biofilm caused a significant enhancement of IL-8 expression, compared to the control group (6.3-fold), which was more potent than the effect caused by the 7-species biofilm lacking the “red complex” species (2.2-fold) (Figure 1). Moreover, the 3-fold difference in IL-8 gene expression observed between the 10-species and the 7-species biofilm-challenged groups also proved to be statistically significant. Hence, the presence of the “red complex” species in the biofilm caused a more potent induction of IL-8 expression, at this early time-point. After 24 h of challenge, the basal IL-8 gene expression levels of the gingival multi-layered epithelia was drastically decreased in all groups, compared to the levels detected at 3 h. Despite the much lower expression at this later time-point, the dynamics of IL-8 regulation were reversed. Compared to the un-challenged control group, IL-8 expression was up-regulated by 2.3-fold and 6.2-fold by the 10-species and the 7-species biofilm, respectively (Figure 1). Yet no statistically significant differences were observed between the experimental groups.

**Figure 2:**
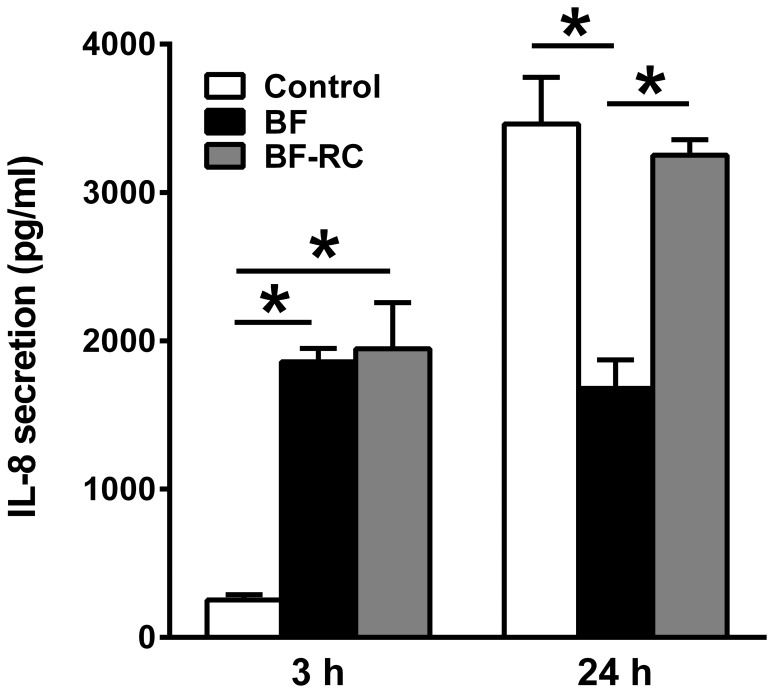
Regulation of IL-8 secretion in response to biofilm challenge. Multi-layered gingival epithelial cell cultures were challenged for 3 h or 24 h with the 10-species or 7-species (excluding *P. gingivalis*, *T. forsythia*, *T. denticola*) subgingival biofilm. IL-8 secretion in the culture supernatant was measured by ELISA. Bars represent mean values ± SEM from three independent cell cultures in each group. One-way ANOVA was used to calculate the differences between groups. Asterisks represent statistically significant differences between groups (*P*<0.05).

The secretion of IL-8 by the cells in response to the two types of biofilms was also investigated after 3 h and 24 h of challenge (Figure 2). During the first 3 h, both biofilms caused a significant induction of IL-8 secretion by the gingival epithelial cultures, which was approximately 7.5-fold higher than the un-challenged control levels. Nevertheless, after 24 h, basal IL-8 secreted levels were elevated more than 10-fold. At this time-point, challenge of the cultures with the 7-species biofilm did not yield any differences in IL-8 secretion, compared to the control group. However, the 10-species biofilm caused a 50% reduction, compared to either the control group, or the 7-species biofilm-challenged group. This indicates that, at this later time-point, the concomitant presence of the three “red complex” species can reduce the levels of secreted IL-8 by the multi-layered gingival epithelial cultures.

## Discussion

Contemporary experimental models of *in vitro* biofilms and their interaction with the host are crucial for the understanding of the pathogenic mechanisms underlying periodontal diseases [17]. The present study investigated the effect of *in vitro* multi-species subgingival biofilms on IL-8 expression and secretion by multi-layered human gingival organotypic epithelia, and evaluated the collective role of the “red complex” species, namely *P. gingivalis*, *T. denticola* and *T. forsythia*, in these effects. The results indicate an early up-regulation of IL-8 gene expression by the biofilms, with the presence of the “red complex”, causing a 3-fold more potent induction. This indicates that these species are potent early inducers of IL-8 transcription, and may aim at driving a fast chemotactic response. In agreement with these findings, single species biofilms of *F. nucleatum*, *A. naeslundii*, *S. gordonii* and *S. oralis* are shown to up-regulate IL-8 gene expression in an oral epithelial cell line after 6 h of challenge, while *Streptococcus sanguinis* did not have any effect, and *P. gingivalis* down-regulated its expression [12]. Nevertheless, these dynamics are reversed after a longer 24 h period of challenge. While the absence of the “red complex” resulted in a higher induction of IL-8 expression, its ubiquitous expression by the gingival epithelial organotypic tissue was dramatically reduced. This transcriptional reduction could well represent a negative feedback mechanism, in an attempt to establish a steady state production of IL-8. Moreover, the increased IL-8 detection in the control group over the experimental period is likely to be attributed to the accumulation of this chemokine in culture, due to its ubiquitous secretion even by healthy epithelium [5]. It should be noted that the control groups included cells cultured in the presence of hydroxyapatite discs pre-treated with saliva, in order to form a pellicle. Although this is an appropriate biological control for the biofilm groups used in this study, the possibility should not be excluded that the salivary proteins alone could in fact alter the expression or secretion of IL-8 by the multi-layered epithelial cell cultures.

The kinetics of IL-8 secretion in the present experimental system appeared considerably different, than those of IL-8 gene expression. When IL-8 secretion by the gingival epithelial culture was considered, this was found to be significantly up-regulated in response to the multi-species biofilm challenge, already after 3 h in culture, irrespective of the presence of the three “red complex” species. After 24 h, IL-8 secretion in the cultures was peaked, compared to the 3 h time-point. However, the levels of IL-8 were reduced to approximately 50% of the control in the presence of the 10-species subgingival biofilm, but were not affected when the three “red complex” species were absent (i.e. 7-species biofilm). Earlier studies, using mono-species biofilms, indicate that after 6 h or 24 h of challenge, *F. nucleatum* and *A. naeslundii* caused a significant increase in IL-8 secretion in oral epithelial cells, whereas *P. gingivalis* inhibited this below control levels and *S. oralis* had no effect [12], [14]. All four of these species are included in the present multi-species biofilm model, and the observed effects are likely to be the net effect of each individual species, as well as the interaction between them. Therefore it is not possible to directly compare the two experimental models. Regarding multi-species biofilms, in a comparable 9-species model (*T. denticola* not included) IL-1, IL-6 and IL-8 secretion were enhanced by gingival epithelial cells at 4 h, followed by a gradual decrease over 24 h [18], a trend similar to the one observed in the present study. In another *in vitro* biofilm model consisting of three species, the combination of *Streptococcus gordonii*, *A. naeslundii* and *F. nucleatum* caused a strong up-regulation IL-8 secretion in by oral epithelial cells, whereas the combination of *S. gordonii*, *F. nucleatum* and *P. gingivalis* lowered or even inhibited the production of IL-8 [16]. This could indicate that the presence of *P. gingivalis*, one of the three “red complex” species, may be involved in the down-play of the chemotactic responses by epithelial cells. Accordingly, the present 10-species subgingival biofilm model has been shown to down-regulate IL-1β expression by gingival fibroblasts, whereas in the absence of *P. gingivalis* this effect was not observed [26].

In conclusion, subgingival biofilms cause an early up-regulation of IL-8 gene expression in multi-layered gingival epithelial cultures, and a late down-regulation of IL-8 secretion, attributed to the presence of the three “red-complex” species. The later effect could constitute either an active down-play of the inflammatory response, or a degradation effect caused by these three highly proteolytic species [19]–[22], [27], [28]. The findings in this complex experimental model are confirmatory the findings of earlier studies using epithelial monolayers or biofilms consisting of fewer bacterial species [11]–[18]. They further support the notion that, as part of polymicrobial communities, *P. gingivalis*, *T. denticola* and *T. forsythia* can cause over time a biphasic regulation of IL-8 chemotactic signals by gingival epithelia. Hence, they hold the potential to disrupt the host-microbial homeostasis, possibly as a strategy to manipulate the local innate immune responses, and enhance their chances of survival within the subgingival niche [4], [29].
